# Network Pharmacology Analysis, Molecular Docking Integrated Experimental Verification Reveal the Mechanism of *Gynostemma pentaphyllum* in the Treatment of Type II Diabetes by Regulating the IRS1/PI3K/Akt Signaling Pathway

**DOI:** 10.3390/cimb46060333

**Published:** 2024-06-01

**Authors:** Songqin Yang, Mao Zhao, Mingxing Lu, Yuhan Feng, Xia Zhang, Daoping Wang, Wenwen Jiang

**Affiliations:** 1 School of Pharmaceutical Sciences, Guizhou University, Guiyang 550025, China; 18311855651@163.com (S.Y.); 17585376169@163.com (M.Z.); 18892455443@163.com (M.L.); fengyuhan0229@outlook.com (Y.F.); zhangxia12121@163.com (X.Z.); 2 Key Laboratory of Natural Products Chemistry, Guizhou Academy of Sciences, Guiyang 550014, China; wdp_7897@aliyun.com

**Keywords:** type 2 diabetes, *Gynostemma pentaphyllum* (Thunb.) Makino, insulin resistance, network pharmacology, molecular docking, PI3K/Akt signaling pathway

## Abstract

*Gynostemma pentaphyllum* (Thunb.) Makino (GP), a plant with homology of medicine and food, as a traditional Chinese medicine, possesses promising biological activities in the prevention and treatment of type 2 diabetes mellitus (T2DM). However, the material basis and the mechanism of action of GP in the treatment of T2DM have not been fully elucidated. This study aimed to clarify the active components, potential targets and signaling pathways of GP in treating T2DM. The chemical ingredients of GP were collected by combining UPLC-HRMS analysis and literature research. Network pharmacology revealed that GP had 32 components and 326 potential targets in treating T2DM. The results showed that GP affected T2DM by mediating the insulin resistance signaling pathway, PI3K/Akt signaling pathway and FoxO1 signaling pathway, which had a close relationship with T2DM. Molecular docking results showed that STAT3, PIK3CA, AKT1, EGFR, VEGFA and INSR had high affinity with the active compounds of GP. In vitro, GP extracts obviously increased the glucose uptake and glucose consumption in IR-HepG2 cells. GP extracts increased the levels of PI3K, p-AKT, p-GSK3β and p-FoxO1 and decreased the expression of p-IRS1, p-GS, PEPCK and G6Pase, which indicated that GP could promote glycogen synthesis and inhibit gluconeogenesis by regulating the IRS1/PI3K/Akt signaling pathway. The results demonstrated that GP could improve insulin resistance by promoting glucose uptake and glycogen synthesis and inhibiting gluconeogenesis through regulating the IRS1/PI3K/Akt signaling pathway, which might be a potential alternative therapy for T2DM.

## 1. Introduction

Diabetes is a very common chronic metabolic disease, which brings serious harm to human health and life quality. The patients with diabetes are getting younger and younger, which has gradually become a hot topic of concern. The latest report indicated that if appropriate measures are not taken, there will be 643 million (11.3%) diabetic patients in the world by 2030, and this number will jump to an astonishing 783 million (12.2%) by 2045 [1]. The number of diabetic patients is increasing rapidly, which has threatened global health and become a severe and urgent challenge. Type 2 diabetes (T2DM) is one of the types of diabetes with high incidence. The etiology of T2DM is mainly related to acquired metabolic disorders and congenital genetic susceptibility. In recent years, with the change in human lifestyle and eating habits, the number of diabetic patients has been increasing obviously [2,3]. Diabetes and its complications seriously endanger human health, so it is imperative to treat T2DM [4,5].

In modern medicine, drugs for treating diabetes mainly include the following categories: insulin sulfonylureas, α-glucosidase inhibitors, insulin sensitizers and insulin secretors [6,7]. However, these drugs are accompanied by many side effects such as hypoglycemia and gastrointestinal reaction. Furthermore, diabetes is a complex disease, and it is impossible to achieve a good therapeutic effect by relying on a single drug corresponding to a single target [8]. Therefore, it is urgent to explore a convenient, nontoxic and safe drug to treat T2DM. In China, people used traditional Chinese medicine to treat and prevent diabetes thousands of years ago [9]. *Gynostemma pentaphyllum* (Thunb.) Makino (GP), a plant with homology of medicine and food, is a traditional national medicine in China. It can be eaten as a vegetable and herbal tea; meanwhile, it has high medicinal value [10]. The Compendium of Materia Medica recorded that it has the efficacy of treating edema, fever, tumor and hematuria [11]. Many bioactive compounds have been identified and isolated from GP, including saponins, polysaccharides, flavonoids and phytosterols [12]. A large number of studies showed that GP has a variety of pharmacological activities such as anti-Parkinson’s disease, anti-atherosclerosis, anti-cancer, and also has the functions of lowering blood sugar [13,14,15]. The ethanol extract of GP could regulate the glucose level by stimulating insulin secretion and inhibiting the activities of α-amylase and α-glucosidase. A recent experimental study demonstrated that GP could significantly improve insulin resistance in patients with T2DM, and its mechanism might be related to regulating glucose metabolism and hypoglycemic effect by active substances of GP [16,17]. Thus, GP extract is usually used as an additional therapy for T2DM. Compared with modern medicine, GP is a traditional herbal medicine with both medicinal and edible properties, which has fewer adverse reactions and lower toxicity and is cheaper and easier to obtain. Therefore, GP has unique advantages and high research value. However, the use and development of GP are limited because of its complexity of ingredients and the ambiguity of its molecular mechanisms.

Network pharmacology is based on the analysis of multiple pharmacology and molecular networks in systems biology. It provides the genetic targets of chemical components, biological targets and biological functions to predict the mechanism of certain compounds in vivo. Network pharmacology is an effective tool to clarify the pharmacological effects of traditional Chinese medicine [18,19].

**Figure 1:**
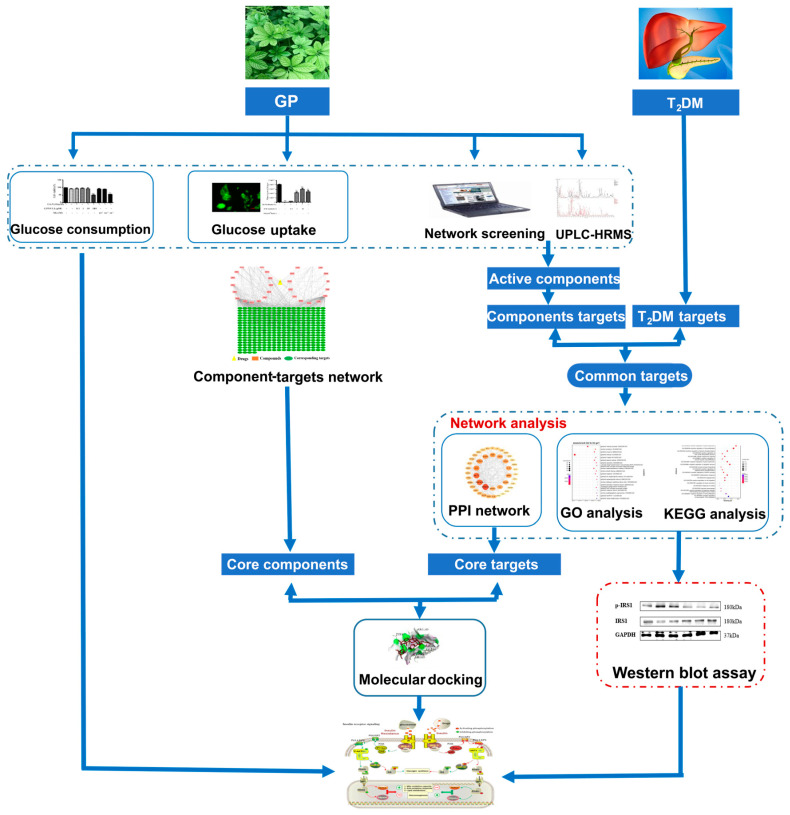
The detailed flowchart of the current study.

Therefore, our study aims to further evaluate the active compounds and the therapeutic potential mechanism of GP in treating T2DM. Firstly, we screened the T2DM-related highlight genes and the effective components of GP by using the network pharmacology. Furthermore, the effective components and core targets were further verified through molecular docking. Finally, pharmacological experiments in vitro proved that GP extract could treat T2DM through the PI3K/Akt/GSK-3β and PI3K/Akt/FoxO1 pathways. It is expected that this study can provide theoretical reference for the study of GP in treating T2DM. The research flow of this study is shown in the figure (Figure 1).

## 2. Materials and Methods

### 2.1. Chemicals and Reagents

Metformin hydrochloride was bought from Beijing Solarbio Technology Co., Ltd. (Beijing, China). HepG2 cells were bought from Cell Bank, Chinese Academy of Sciences (Shanghai, China). RIPA lysis buffer was obtained from Cowin Biotech Co., Ltd. (Taizhou, China). IRS-1 and p-IRS-1, Akt and p-Akt, and GAPDH were bought from Affinity Bioscience (Cincinnati, OH, USA). PI3K was acquired from ImmunoWay Biotechnology Company (Plano, TX, USA). GSK-3β and p-GSK-3β, GS and p-GS, FoxO1 and p-FoxO1, PEPCK and G6Pase were obtained from PEPROTECH (Rocky Hill, NJ, USA). A glucose-determining kit was purchased from Rong Sheng Biotech Co., Ltd. (Shanghai, China). Glucosamine was acquired from Beyotime Biotechnology Co., Ltd. (Shanghai, China). An HRP substrate kit was bought from Millipore Corporation (Bellerica, MA, USA). The oil red O stain kit was bought from Beyotime Biotechnology (Shanghai, China). Methanol (≥99.5%, CAS:67-56-1) was bought from Chuan Dong Chemical (Group) Co., Ltd. (Chongqing, China). Ethanol (≥99.7%) was purchased from Tianjin Comio Chemical Reagent Co., Ltd. (Tianjin, China).

### 2.2. Extraction and UPLC-HRMS Analysis of Compounds from GP

The dried GP was bought from Health home pharmaceutical Co., Ltd. (Guiyang, China). It was identified as *Gynostemma pentaphyllum* (Thunb.) Makino (GP) by Professor Jiang Wenwen of Guizhou University, Guizhou, China. The dried GP was extracted with 75% ethanol at 80 °C three times, each time for 2 h. The GP extract samples were combined, then filtered and concentrated. Finally, the extract was prepared into a solution of 100 mg/mL.

A Thermo Scientific Q Exactive Focus system (Thermo Fisher Scientific, Waltham, MA, USA) and a Dionex Ultimate 3000 RSLC system (HPG) were used to qualitatively analyze the GP extract. Chromatographic conditions: chromatographic column: ACE Ultracore 2.5 Super C18 (100 mm × 2.1 mm, Philomen, Guangzhou, China). The flow rate: 0.30 mL/min. Mobile phase B: 0.1% formic acid aqueous solution; Mobile phase C: Acetonitrile (containing 0.1% formic acid). The elution process is: 0–2 min, 95% B; 3–42 min, 95% B; 42–47 min, 5% B; 47–47.1 min, 5%–95% B; 47.1–50 min, 95% B. The injection volume of the sample was 10 μL, and the column temperature: 40 °C.

Mass spectrometry conditions: The ion source was HESI-II. We performed component detection in positive and negative ion modes. Spray voltage: 3000 V; Sheath gas pressure: 35 arb. Auxiliary flow: 10 arb. Exhaust flow: 10 arb; Spray voltage: 2500 V. The mass scanning range of primary mass spectrometry was *m*/*z* 100–1500, and the spray voltage was 3.0 kV (+)/2.5 kV (−). Ion transfer tube temperature: 320 °C.

### 2.3. Active Compounds Screening and Targets Fishing

In this study, “Gynostemma pentaphyllum” was the keyword to seek the active compounds of GP using the TCMSP database (https://old.tcmsp-e.com/tcmsp.php) (accessed on 23 April 2022) by setting the ADME parameter screening conditions: drug similarity (DL) ≥ 0.18, oral absorption utilization (OB) ≥ 30%. All targets of core components were screened from the TCMSP database and Swiss TargetPrediction database (http://swisstargetprediction.ch/) (accessed on 27 April 2022).

### 2.4. Construction and Analysis of Drug–Component–Target Interaction

Cytoscape is a useful software which provides greater flexibility in network analysis, import and data visualization [20]. The collected disease targets and the targets of active ingredient in GP were imported into the Cytoscape 3.7.2 software and we obtained the drug–ingredient–target interaction network through processing. The edges represented the correlation among drugs, components and targets, and the nodes represented components, drugs or targets. Topological parameters calculated by Network Analyzer were used to screen out the import targets and components. The greater the absolute value, the more likely these components were to be the key components of GP.

### 2.5. Venny Analysis

In the GeneCards database (https://www.genecards.org/) (accessed on 1 May 2022), “Type 2 Diabetes Mellitus” was used as the keyword. In addition, the target with a correlation score ≥ 50 was selected as an important target related to diabetes. The targets of type 2 diabetes and active components were analyzed by the Venny tool (https://bioinfogp.cnb.csic.es/tools/venny/) (accessed on 1 May 2022), and the common targets were regarded as the critical targets of GP in treating type 2 diabetes.

### 2.6. Construction of the PPI Network

The STRING database (https://cn.string-db.org/) (accessed on 1 May 2022) could be used to analyze the interaction between protein and protein, which could automatically generate the interaction network. The obtained intersection targets were input into the STRING database to obtain the protein–protein interaction (PPI) network. The obtained PPI network information was imported into Cytoscape 3.7.2 software for refinement and visualization. Then, by analyzing the network topology parameters, the key targets were selected according to the degree value ranking. The circular nodes represented the targets, and nodes changed from small to large. In PPI networks, nodes with larger degree values usually play a more important role.

### 2.7. Functional Enrichment Analysis

The DAVID database (https://david.ncifcrf.gov/) (accessed on 2 May 2022) and OmicShare (https://www.omicshare.com/) (accessed on 2 May 2022) were used to enrich and analyze the gene ontology (GO) and Kyoto Encyclopedia of Genes and Genomes (KEGG) involved in the predicted targets. The *p* value was set at *p* < 0.05. Then, GO analysis and KEGG pathway analysis were visualized by online drawing software bioinformatics.

### 2.8. Component–Target Molecular Docking

The binding affinity between macromolecular receptors and ligands was predicted by using molecular docking. In recent years, molecular docking has become one of the essential methods to screen active ingredients of TCM [21]. And it has developed into a formidable tool for drug development [22]. Therefore, in this study, Discovery Studio software version 1.5.6 was used for molecular docking between core ingredients of GP and core targets of GP in treating T2DM. All the compound structures were drawn by ChemDraw version 15.0 and the crystal structure of the key targets were obtained from the Uniprot database (https://www.uniprot.org/) (accessed on 5 May 2022). According to the results of the molecular docking score, the effective components with good binding ability to the core target proteins could be evaluated. The active components of GP were docked with the insulin receptor (INSR) through Discovery Studio software version 15.0. The active ingredients with good stimulation scores were screened out based on the docking results [23].

### 2.9. Cell Culture

HepG2 cells were acquired from the Cell Bank of the Chinese Academy of Science (Shanghai, China). Multiple studies have shown that human liver cancer cell line HepG2 cells were often used to study the pathogenesis of insulin. It is reported that the model of inducing insulin resistance in HepG2 cells usually uses 18 mM of glucosamine (Glu) for 18 h [24]. Therefore, HepG2 cells were induced by 18 mM of glucosamine for 18 h to establish the model of insulin resistance in this study.

### 2.10. Cytotoxicity Was Detected by MTT Assay

MTT assay was used to detect the effects of GP extract on the viability of HepG2 cells. The prepared HepG2 cells suspension was cultured in 96-well plates with a density of 1 × 10^4^ cell/mL. When the cells in the plate grew to about 80%, 18 mM of GlcN was added to induce the insulin resistance (IR) model for 24 h. Then, the medium in the well was removed, and concentrations of GP extract (0.1, 1, and 10 µg/L) were added to each well for 24 h. After treatment, we added the prepared MTT solution into each well for 4 h. Subsequently, the liquid in the wells was removed, and 100 μL of DMSO was added. Finally, the wavelength of the microplate reader was set at 490 nm, and the absorbance was measured.

### 2.11. Glucose Consumption Assay

HepG2 cells culture was the same as Section 2.12. Different concentrations of GP extract (0.1, 1, and 10 µg/L) were added to each well for 24 h. After 24 h, a glucose reagent was added for 15 min. After treatment, the culture medium was collected. After 24 h, glucose reagent was added. Finally, the glucose concentrations in the supernatant could be obtained by measuring the OD values at the wavelength of 505 nm.

### 2.12. 2-NBDG Glucose Uptake Assay

Fluorescence 2-NBDG was used to evaluate the glucose uptake level of cells. HepG2 cells (1 × 10^5^ cell/well) were cultured in a six-well plate. When the cells in the plate grew to about 80%, 18 mM of GlcN was added to induce the insulin resistance (IR) model for 24 h. Different concentrations of GP extract (0.1, 1, and 10 µg/L) were added to each hole for 24 h. Subsequently, 2-NBDG (100 μM) was added to the well plate for 1 h. After treatment, the cells were slowly washed twice with PBS to remove residual probes. The orifice plates were photographed under the inverted fluorescence microscope, and the results were analyzed by Image J version 1.4.3 analysis software.

### 2.13. Western Blot Assay

Cells were treated with metformin (10^−4^ M) and GP extract (0.1, 1, and 10 µg/mL) for 1 h, and the cells were lysed with RIPA buffer. After 20 min, the collected cell mixture was centrifuged at 4 °C, 12,000 r/min for 15 min. And then the protein concentration in the supernatant was determined by BCA kit. We added a quarter of buffer to protein solution and denatured at 80 °C for 8 min. Protein samples were separated by SDS-polyacrylamide gel with a concentration of 12.5%, and then the protein on the gel was transferred to PVDF membrane. The rapid blocking solution was added for 30 min, and then we washed the membranes with TBST. Subsequently, the membranes were incubated with specific primary antibody. After incubation for 24 h, the membranes were washed with TBST and the HRP-labeled secondary antibody was diluted according to the ratio of 1:10,000 and transferred to the antibody incubator with membrane for 1 h. Finally, the membranes were treated by chemiluminescence instrument, and the images obtained were quantitatively analyzed by Image J analysis version 1.4.3 software.

### 2.14. Statistic Analysis

GraphPad Prism version 7.0, Student *t* test or ANOVA were used for statistical analysis to evaluate statistical significance. Significance was defined as *p* < 0.05. All experiments were carried out at least three times.

## 3. Results

### 3.1. The Screening of Active Compounds and Targets Fishing

Based on network pharmacology, UPLC-HRMS analysis and literature research, 32 compounds with potential biological activities were screened out. The details of the active ingredients are shown in Table 1. The corresponding targets of the compound were searched using the Swiss TargetPrediction database, and 326 targets were obtained.

### 3.2. Construction and Analysis of Drug–Component–Target Interaction

**Figure 2:**
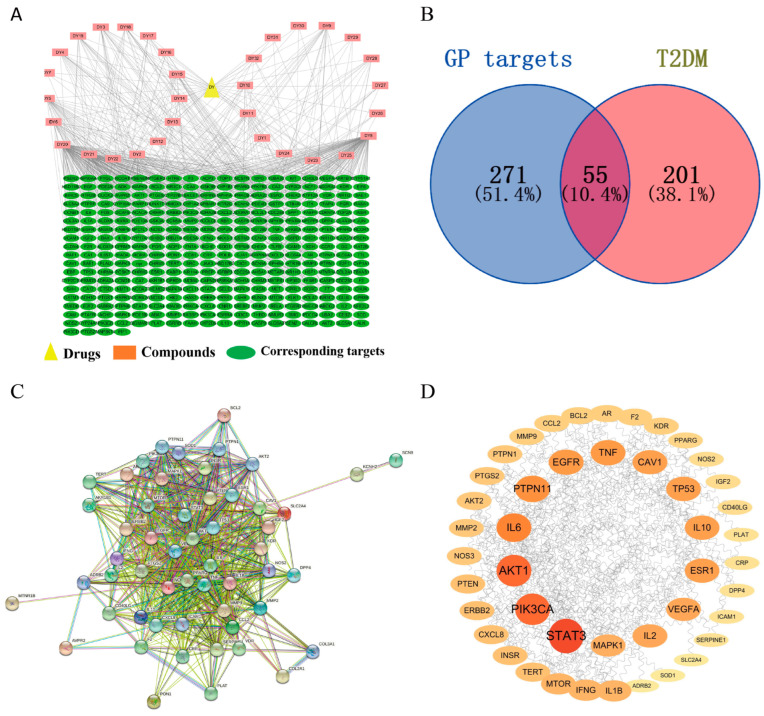
Network pharmacology prediction results. (**A**) Network diagram of drug–component–target; (**B**) Venn diagram of GP and T2DM intersection targets; (**C**) Protein–protein interaction network obtained from the STRING database; (**D**) the PPI network of the anti-T2DM targets of GP embellished by Cytoscape 3.7.2.

The interactive network graph consists of 358 nodes and 1749 edges (Figure 2). The 358 nodes in the figure represent 32 active ingredients and potential targets of GP in treating T2DM, which are distinguished by different colors. Furthermore, the 1749 edges describe the interaction between these nodes. Each compound linked four or more targets. Most of the degree values were greater than 20, which indicated that these compounds were the core compounds of GP in the treatment of T2DM.

### 3.3. Prediction of Gene Targets of T2DM

A total of 14,330 T2DM-related targets were obtained by keyword search. According to the median score, important targets were screened. The targets with a score greater than 27.495 were regarded as the potential targets of T2DM, and finally 256 targets related to T2DM were screened out. By crossing the targets of the active components in GP with the potential targets of T2DM, 55 potential targets of GP in treating T2DM were obtained, as shown by a Venn diagram (Figure 2).

### 3.4. Construction of the PPI Network

The 55 overlapping targets obtained were regarded as potential targets for GP in treating T2DM. As shown in Figure 2, there are 54 core targets and 1027 in the PPI network. Then, the PPI network was embellished by Cytoscape 3.7.2. The nodes represent the target proteins, and the larger the node, the greater the degree value. In PPI networks, nodes with larger degree values usually play a more important role. Among the top 20 core targets, 5 targets (STAT3, PIK3CA, AKT1, EGFR and VEGFA) were closely related to diabetes (Figure 2).

### 3.5. GO and KEGG Enrichment Analysis

**Figure 3:**
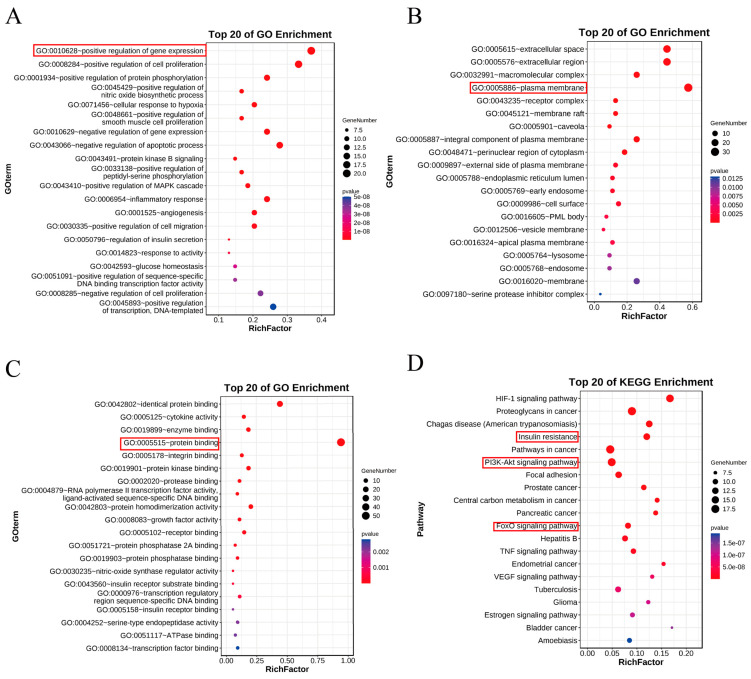
GO and KEGG enrichment analysis. (**A**) Biological Processes (BP); (**B**) Cellular Component (CC); (**C**) Molecular Function (MF); (**D**) KEGG enrichment analysis.

According to the *p* value less than 0.05, 464 GO terms were obtained, which included 49 cellular component terms, 43 molecular functions and 372 biological processes. The top 20 items with the lowest *p* value were selected for GO analysis, and the secondary classification histogram and functional information were drawn by using the OmicShare tools. The biological processes’ results showed that the targets mainly included positive regulation processes gene expression, positive regulation of cell proliferation, PKB(AKT) signaling pathway and insulin secretion. The terms of cell components included plasma membrane, extracellular space, receptor complex and so on. The molecular function terms included protein recognition binding, cytokine activity, insulin receptor binding, insulin receptor substrate binding and so on (Figure 3). According to the *p* value less than 0.05, 143 KEGG path items were obtained. The 20 pathways with the highest *p* value were selected, and we drew the bubble charts with the OmicShare tools. The results showed that the pathways mainly included insulin resistance, cancer pathway, PI3K-Akt signaling pathway, FOXO signaling pathway, TNF signaling pathway, and VEGF signaling pathway (Figure 3).

### 3.6. Molecular Docking Studies of Active Components with Key Targets

**Figure 4:**
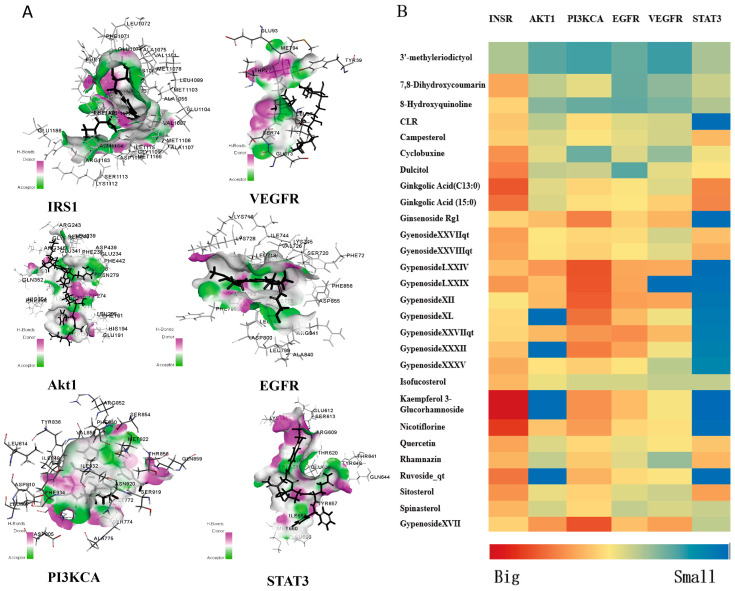
Molecular docking of active ingredients and key targets. (**A**) Docking conformation of Gypenoside XVII and STAT3, PIK3CA, AKT1, EGFR and VEGFA; (**B**) Heat map showing the docking scores of STAT3, PIK3CA, AKT1, EGFR, VEGFA and INSR with the core components of GP.

Signal transducer and activator of transcription 3 (STAT3), Phosphatidylinositol 4,5-bisphosphate 3-kinase catalytic subunit alpha isoform (PIK3CA), RAC-alpha serine/threonine-protein kinase (AKT1), Epidermal growth factor receptor (EGFR), Vascular endothelial growth factor A (VEGFA), insulin receptor (INSR) and 28 core components of GP were selected to conduct molecular docking by using Discovery Studio (DS) software. Furthermore, the compounds with good binding ability to core protein were screened out from these compounds. The molecular docking results were gained from the compound–target complexes of these compounds targeting STAT3, PIK3CA, AKT1, EGFR, VEGFA and INSR. It showed that key protein INSR had strong binding ability to nine components, including Ginkgolic acid (GA13:1), Ginkgolic acid (GA15:1), Gypenoside XXVII, Gypenoside XXVIII, Hesperetin, Nicotiflorin, Quercetin, Spinasterol and Gypenoside XVII (Figure 4). Among them, Gypenoside XVII could bind to six core proteins well, and its docking scores were high among these compounds. Gypenoside XVII, as one of the core compounds of GP, has not been reported in the treatment of diabetes.

### 3.7. Effect of GP Extract on Glucose Consumption in IR HepG2 Cells

**Figure 5:**
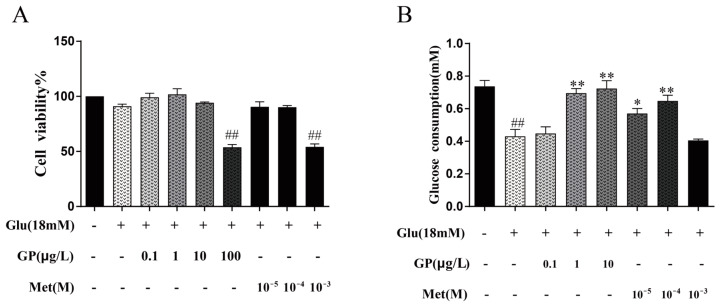
Effects of GP extract on the glucose consumption in insulin-resistant hepatocytes. (**A**) Effects of GP extract in different concentrations on the proliferation of HepG2 cells. (**B**) Effects of GP extract on glucose consumption in insulin-resistant hepatocytes. Data are expressed as mean ± S.D. For each group, n = 3. * *p* < 0.05, ** *p* < 0.01 vs. IR group (18 mM of GlcN stimulated). ^##^ *p* < 0.01 vs. normal group.

MTT assay results showed that glucosamine (18 mM) had no significant toxicity in insulin-resistant HepG2 cells. After comparing the metformin group (lower than 10^−3^ mol/L) with the control group, it showed that metformin had no toxicity on HepG2 cells. However, when the concentration of metformin hydrochloride was 10^−3^ mol/L, the results showed that it had obvious toxicity to cells. Therefore, in the subsequent studies, metformin hydrochloride with a concentration of 10^−4^ mol/L was selected as the positive control group. Furthermore, the MTT toxicity assay showed that GP extract (0.1, 1, and 10 µg/L) had no significant effect on cells (Figure 5).

The potential effects of GP extract on glucose consumption of HepG2 cells were evaluated. In the treatment group with only 18 mM of GlcN, the glucose consumption of HepG2 cells decreased rapidly. After treating with GP extract, the glucose consumption increased in a concentration-dependent manner (Figure 5). The results showed that GP extract could improve glucose consumption in HepG2 cells with insulin resistance.

### 3.8. GP Extract Increased Glucose Uptake in Glucosamine-Induced HepG2 Cells

**Figure 6:**
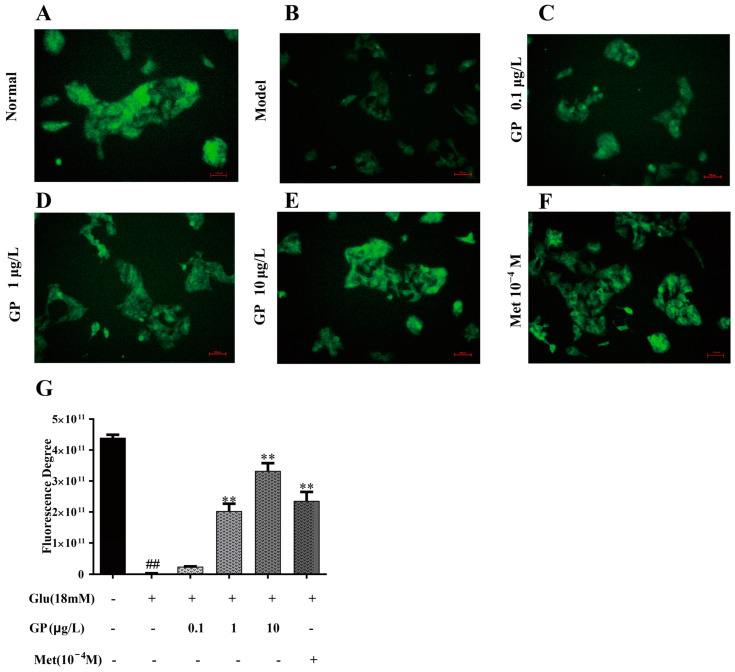
The fluorescence results of 2-NBDG glucose uptake. (**A**) Normal control group; (**B**) Model group (IR group, 18 mM of GlcN stimulated); (**C**–**E**) Containing different concentrations of GP extract; (**F**) metformin control group; (**G**) Column chart of glucose uptake. 100× magnification. n = 3 for each group. Values are mean ± SD. ** *p* < 0.01 vs. IR group (18 mM of GlcN stimulated). ^##^ *p* < 0.01 vs. normal group.

The potential effects of GP extract on glucose uptake of IR-HepG2 cells were evaluated. The IR-HepG2 cells in the well plate were treated with GP extract (0.1, 1, 10 µg/mL) and the fluorescent indicator 2-NBDG was added to the incubated cells for 60 min. The area and fluorescence density of IR-HepG2 cells treated with GlcN (18 mM) decreased rapidly compared with the normal group, which indicated that the glucose uptake in IR-HepG2 decreased significantly (Figure 6) However, GP extract could significantly increase the glucose uptake in IR-HepG2 in a concentration-dependent manner. The results showed that GP extract could promote glucose uptake of IR-HepG2 cells.

### 3.9. Effect of GP Extract on the IRS-1/PI3K/AKT Signaling Pathway in Glucosamine-Induced HepG2 Cells

Insulin interacts with INR to initiate the signaling pathway of insulin and improve insulin resistance. The tyrosine of IRS family members was phosphorylated by the activated receptor, and the PI3K/Akt pathway was activated [25]. The PI3K/Akt signaling pathway was of great significance to metabolism; this pathway and related molecules were considered to be important parts of treating T2DM [26]. In addition, according to the results of network pharmacology, the PI3K/Akt signaling pathway might be the key way for GP extract to treat T2DM. Therefore, it was necessary to further study whether GP extract affected the PI3K-Akt signaling pathway.

**Figure 7:**
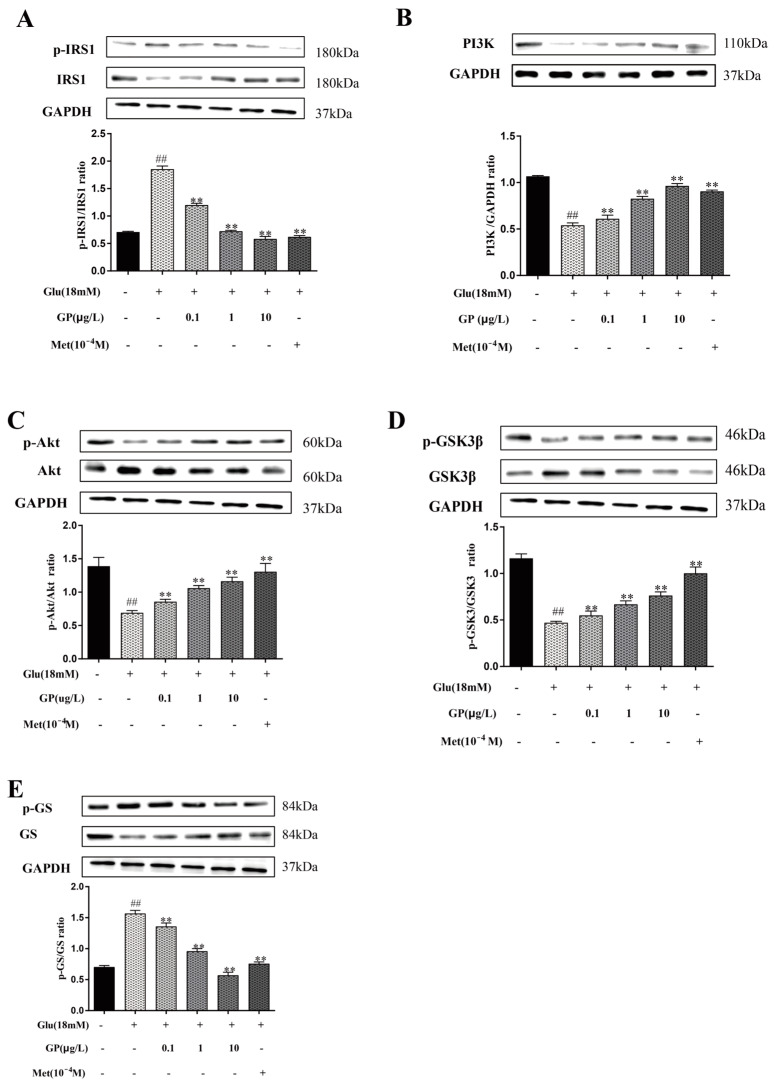
Effects of GP extract on glycogen synthesis in IR-HepG2 cells. (**A**) The expression levels of IRS1, p-IRS1 and the ratios of P-IRS-1/IRS-1; (**B**) The expression levels of PI3K and GAPDH were used as a loading control; (**C**) The expression levels of Akt, p-Akt, and the ratios of p-Akt/Akt; (**D**) The expression levels of GSK-3β and p-GSK-3β and the ratios of p-GSK-3β/GSK-3β; (**E**) The expression levels of GS, p-GS, and the ratios of p-GS/GS. Data are expressed as mean ± S.D. For each group, n = 3. ** *p* < 0.01 vs. IR group (18 mM of GlcN stimulated). ^##^ *p* < 0.01 vs. normal group.

Both IRS-1 and PI3K were reduced (Figure 7) in the IR-HepG2 cells treated with glucosamine (*p* < 0.01), but GP extract prevented the decrease of two proteins. In addition, the expression of p-Akt in HepG2 cells treated with glucosamine was decreased obviously, but GP extract improved this situation in a concentration-dependent manner (*p* < 0.05). Glycogen synthase (GS) is a key enzyme to regulate glycogen synthesis, which catalyzes the rate-limiting steps of glycogen synthesis. The report revealed that insulin could increase the phosphorylation of GSK-3β, which activated GS and promoted glycogen synthesis [27]. Glucosamine treatment reduced the phosphorylation of GSK-3β and up-regulated the expression of p-GS, but all of these were improved by GP extract in HepG2 cells (*p* < 0.05). These findings indicate that GP extract could up-regulate glycogen synthesis in IR-HepG2 cells through the PI3K/Akt/GSK-3β pathway.

### 3.10. Effect of GP Extract on the Expression of G6Pase and PEPCK in IR-HepG2 Cells

**Figure 8:**
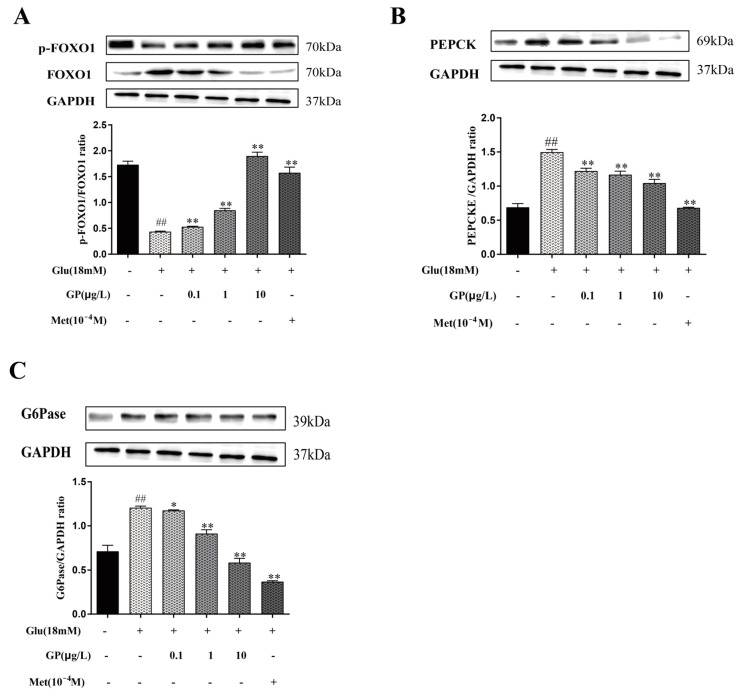
Effect of GP extract on gluconeogenesis in IR-HepG2 cells via the PI3K/Akt/FoxO1 pathway. (**A**) The protein expression levels of p-FoxO1 and FoxO1, and the ratios of p-FoxO1/FoxO1; (**B**) The protein expression levels of PEPCK, the ratios of PEPCK/GAPDH; (**C**) The protein expression levels of G6Pase, the ratios of G6Pase/GAPDH. Data are expressed as mean ± S.D. For each group, n = 3. * *p* < 0.05, ** *p* < 0.01 vs. IR group (18 mM of GlcN stimulated). ^##^ *p* < 0.01 vs. normal group.

Activation of FoxO1 by phosphorylation promotes the expression of PEPCK and G6Pase in hepatocytes, which can restrain hepatic gluconeogenesis [28]. The results (Figure 8) showed that the expression of p-FoxO1 was reduced in IR HepG2 cells (*p* < 0.01), but GP extract could improve this expression. In addition, the treatment of glucosamine increased the expression of PEPCK and G6Pase in HepG2 cells. After treating with GP extract, the expression of these two proteins decreased significantly (*p* < 0.01). The results indicated that GP extract rectified the enhanced glucose production in HepG2 cells with IR.

## 4. Discussion

T2DM is a common chronic metabolic disease that is usually accompanied by different complications [29]. The increasing prevalence of type 2 diabetes, insulin resistance and obesity has become a public health problem and increased the economic burden for the global healthcare system [30]. The control and treatment of type 2 diabetes is imminent, and there are quite a few international guidelines and institutions for the improvement of diabetes [31,32,33]. The efficacy of medicinal herbs and medical plant compounds on T2DM treatment have been highlighted in multiple studies [34,35,36].

GP has been widely used as medicine and tea in the history of ethnic medicine and botany in China. The medicinal uses and precautions of GP are also recorded in the medical book. According to records, its main functions include detoxification, tonifying deficiency and so on. Recent studies demonstrated that GP could significantly improve insulin resistance in patients with T2DM, and GP extracts are usually used as an additional therapy for T2DM [37,38]. Song et al. found that two saponins extracted from GP improved insulin resistance and hyperglycemia induced by STZ in T2DM mice, which could be achieved through the AMPK-mediated signal pathway [39]. Moreover, Chen et al. found that oral gypenoside tablets combined with repaglinide can significantly improve blood sugar level and reduce myocardial damage and inflammatory effects in patients with type 2 diabetes [40]. Many studies on GP in vivo and in vitro show that GP has hypoglycemic activity and good clinical efficacy [16,41,42], but the hypoglycemic mechanism of GP has not been fully explained. GP is a natural Chinese herbal medicine with the characteristics of multi-components and multi-targets. A variety of active ingredients in the extract of traditional Chinese medicine may have a synergistic effect, which can enhance each other’s effects. Therefore, it is of great significance and value to study the molecular mechanism of the hypoglycemic effect of GP extract. The results of molecular docking showed that the nine active components have good anti-sugar potential, which can also provide reference and ideas for the following research, so as to mine more natural compounds with better effects. Network pharmacology and in vitro experiments explore the molecular mechanism of GP on the molecular level, which can provide a basis for clinical and animal research of GP, clarify the hypoglycemic effect of GP more comprehensively and improve the development and utilization of GP.

The potential molecular mechanisms of GP in treating T2DM were predicted and elucidated by systematic pharmacological method. It was found that 32 active compounds had the therapeutic effect of GP on T2DM. This showed that GP was multi-component and multi-target. After screening and prediction, 54 overlapping targets were found from the targets related to diabetes and GP compounds. The results of the PPI network showed that the top 20 overlapping targets included STAT3, PIK3CA, AKT1, INSR, EGFR and VEGFA. Multiple reports showed that STAT3, PIK3CA, AKT1, INSR, EGFR and VEGFA are primarily involved in T2DM diseases, which suggested that GP may have an anti-T2DM effect. Furthermore, the primary core targets (STAT3, PIK3CA, AKT1, INSR, EGFR and VEGFA) were verified by molecular docking. All these results sufficiently showed that GP had anti-T2DM activity. The results of molecular docking showed that nine active components had good binding ability with key protein INSR, including Ginkgolic acid (GA13:1), Ginkgolic acid (GA15:1), Gypenoside XXVII, Gypenoside XXVIII, Hesperetin, Nicotiflorin, Quercetin, Spinasterol and Gypenoside XVII. Recent studies show that hesperidin, quercetin and spinach sterol all have obvious therapeutic effects on diabetes [43,44,45], which further verifies the accuracy of screening active compounds by network pharmacology and molecular docking. In particular, we found that Gypenoside XVII could bind to six core proteins strongly, and the docking scores were high among the screened compounds. In addition, Gypenoside XVII has been demonstrated to have various pharmacological properties against cerebrovascular, cardiovascular and skin diseases [46]. Therefore, we speculated that Gypenoside XVII might be a key component of GP with anti-diabetic activity and may be a potential candidate for anti-T2DM treatment, which is worthy of further study.

GO enrichment results suggested that GP regulated various biological processes, such as gene-positive regulation, insulin secretion, insulin receptor binding, insulin receptor substrate binding and so on, most of which were closely related to T2DM. Furthermore, according to the analysis result of KEGG enrichment, several signaling pathways related to T2DM (such as the insulin signaling pathway, FOXO signaling pathway, TNF signaling pathway, VEGF signaling pathway and PI3K/Akt signaling pathway) were identified. It indicated that GP played an important role in hyperglycemia through multiple targets and signaling pathways related to T2DM. Among them, the PI3K-Akt signaling pathway is involved in the regulation of various biological activities, which is a classic diabetes pathway with multiple diabetes-related targets. Moreover, when the PI3K-Akt signaling pathway was activated, it could improve sensitivity of cells to insulin, thus regulating glucose metabolism [47]. Thus, the PI3K-Akt signaling pathway was selected for the experimental verification of the follow-up mechanism. Network pharmacology can systematically reveal the mechanism of action of Chinese herbal medicine at the molecular level, which provides effective basis and evidence for supporting the role of GP in type 2 diabetes. However, there are still some limitations in network pharmacology, and its database records genes, proteins and drugs incompletely. At present, no suitable calculation software has been developed [48]. On the whole, network pharmacology is a reasonable way to predict the mechanism of GP in treating T2DM.

The liver is the main organ that reduces insulin sensitivity and glucose tolerance. In the state of insulin resistance, the synthesis of hepatic glycogen decreases and the production of fat increases, which leads to the development of hyperglycemia and T2DM [49]. More than 90% of endogenous glucose in the body is produced by the liver, so inhibiting the production of glucose in the liver is a strategy to T2DM. HepG2 cells were induced by 18 mM of glucosamine for 18 h, and the insulin resistance model was established to explore the effect of GP on insulin resistance cells. This study revealed that GP extract could improve glucose consumption of IR-HepG2 cells in a concentration-dependent manner. The results of 2-NBDG assay demonstrated that GP extract significantly increased glucose uptake of insulin-resistant HepG2 cells. It indicated that GP played an important role in alleviating hyperglycemia and insulin resistance in HepG2 cells. Finally, the mechanism of GP extract in treating diabetes was further verified by Western blot assay.

The PI3K/Akt pathway is the main signaling pathway in responding to insulin, which plays a central role in glucose homeostasis [50,51]. Insulin receptor substrate (IRS1) is a key signal molecule connecting intracellular and extracellular insulin signaling pathways, which is closely related to the physiological effects of insulin. When insulin binds to surface receptors, insulin receptor substrate 1 (IRS1) is phosphorylated at tyrosine sites to inhibit its serine, thus activating the downstream signaling pathway to play a physiological role. However, in the context of insulin resistance, the downstream signaling pathway is suppressed owing to the enhanced phosphorylation of IRS1 serine [52,53]. The results showed that the expression of p-IRS1 in the insulin resistance group was increased significantly compared with that in the control group. After treatment with GP extract, the expression of p-IRS1 in IR-HepG2 cells decreased, especially at a high dose. The downstream PI3K/Akt signaling pathway was activated, resulting in the up-regulation of the expression levels of PI3K, p-Akt, p-GSK3β and GS. These suggested that GP played a critical part in the activation of the ISR1/PI3K/Akt signaling pathway. Akt, as a downstream effector of IRS1 and a key intersection in the PI3K/Akt pathway, plays a crucial role in glucose metabolism. It can regulate various signaling pathways and proteins, including GSK-3β [54], which modulates GS activity. When PI3K/Akt is activated, it triggers Akt phosphorylation, subsequently inhibiting GSK-3β expression, stimulating glycogen synthesis and ultimately exerting hypoglycemic effects [55]. According to this research, it was found that GP extract could increase glycogen synthesis in insulin-resistant HepG2 cells through PI3K/Akt/GSK-3β-mediated GS activation.

**Figure 9:**
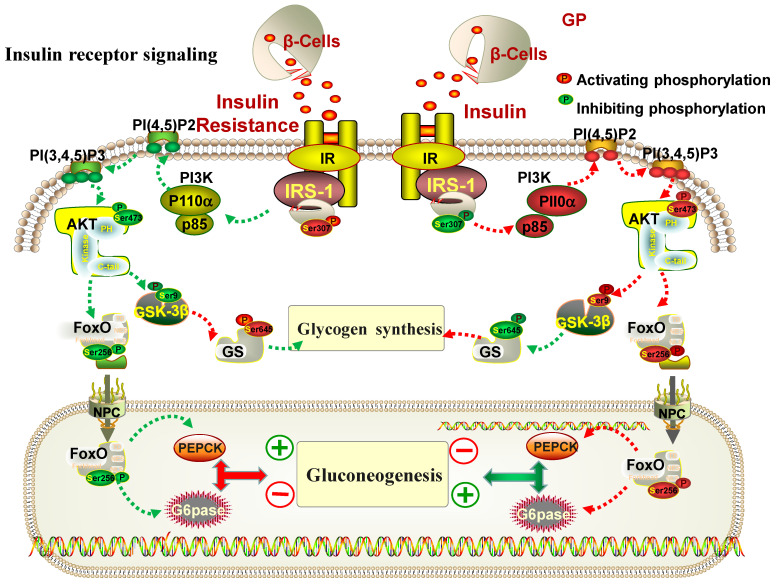
The pharmacological mechanisms of GP extract in the IRS1/PI3K/Akt signaling pathway. GP balanced blood glucose through the PI3K/Akt/GSK-3β and PI3K/Akt/FoxO1 pathways. The red and green lines indicate inhibition and activation, respectively.

It is reported that glucose production in liver is mainly regulated by PEPCK and G6Pase, which are the crucial target genes of FoxO1 and key enzymes in catalyzing gluconeogenesis [56]. When FoxO1 is activated, gluconeogenesis is induced by increasing the expression of PEPCK and g6p enzymes. Insulin can inactivate the phosphorylation of FoxO1 by activating the PI3K/Akt pathway, thus inhibiting hepatic gluconeogenesis [57]. Our results showed that the protein expressions of PEPCK, G6Pase and FoxO1 were increased obviously in HepG2 cells with insulin resistance, whereas GP extract could significantly restore their expressions. It indicated that GP extract could activate insulin receptor-mediated signaling to regulate the gluconeogenesis and the balance of blood sugar. The pharmacological mechanisms of GP extract in the IRS1/PI3K/Akt signaling pathway are shown in Figure 9.

The PI3K/Akt signaling pathway plays a key role in cell homeostasis and metabolism, such as glucose homeostasis, lipid metabolism and protein synthesis [58,59]. Because of its multiple functions, its imbalance is closely related to the development of T2DM and metabolic syndrome related to T2DM. On the one hand, PI3K/Akt regulates glucose metabolism through FoxO1 and GSK-3β. On the other hand, FoxO1 simultaneously activates Akt to increase energy production and inhibits mTORC1 to reduce the production of lipids and protein [60]. The activation of the PI3K/AKT signaling pathway can significantly improve various abnormal indexes of T2DM in vivo and in vitro, regulate the metabolic levels of glucose and lipid protein in insulin target organs and cells, improve insulin resistance to some extent and play a hypoglycemic role. In the present study, GP extract regulated the PI3K/Akt pathways in insulin-resistant HepG2 cells, and it indicated that GP might also regulate the lipid metabolism and protein synthesis.

According to the traditional Chinese medicine (TCM) theory, GP, a heat-clearing and drug detoxifier in TCM, has a cold nature with a slightly bitter taste. Therefore, long-term drinking of GP tea will stimulate the gastrointestinal tract, prompting excessive secretion of stomach acid, resulting in gastrointestinal digestive function abnormalities. However, because of the immeasurable potential of GP in the treatment of chronic diseases, and because of the homology of GP with food and drugs, the metabolites of GP often have fewer side effects and higher clinical efficacy. Therefore, we firmly believe that GP is about to make a new breakthrough in the treatment of T2DM.

## 5. Conclusions

In summary, this study used UPLC-HRMS, network pharmacology, molecular docking and experimental verification to reveal the effective substances and potential molecular mechanism of GP in the treatment of T2DM. GP extract could play a role in treating T2DM through the IRS1/PI3K/Akt signaling pathway. The results showed that GP extract obviously increased the absorption and consumption of glucose in hepatocytes with insulin-resistant (IR). Moreover, GP extract could increase glycogen synthesis through GS activation mediated by the PI3K/Akt/GSK-3β pathway and reduce gluconeogenesis through inhibition of the expression of PEPCK and G6pase mediated by the PI3K/Akt/FoxO1 pathway to improve glucose homeostasis. GP regulated hepatic glucose metabolism through these three pharmacological pathways, which might be one of the molecular mechanisms of GP in treating T2DM.

## Figures and Tables

**Table 1:** The characteristics of the 32 active compounds of GP.

NO	Molecular Name	CAS	OB (%)	DL	Molecular Weight
DY1	Homoeriodictyol	446-71-9	51.61	0.27	302.28
DY2	Rhamnazin	552-54-5	47.14	0.34	330.31
DY3	Sitosterol	68555-08-8	36.91	0.75	414.79
DY4	Ruvoside_qt	6859-20-7	36.12	0.76	390.57
DY5	Spinasterol	481-18-5	42.98	0.76	412.77
DY6	Campesterol	474-62-4	37.58	0.71	400.76
DY7	Isofucosterol	481-14-1	43.78	0.76	412.77
DY8	Ginsenoside f2	62025-49-4	36.43	0.25	785.14
DY9	CLR	80356-14-5	37.87	0.68	386.73
DY10	Quercetin	73123-10-1	46.43	0.28	302.25
DY11	Cyclobuxine	2241-90-9	84.48	0.70	386.69
DY12	Gypenoside LXXIV	110261-97-7	34.21	0.24	801.14
DY13	Gypenoside LXXIX	110282-46-7	37.75	0.25	785.14
DY14	Gypenoside XII	80321-64-8	36.43	0.25	785.14
DY15	Gypenoside XI	94987-10-7	4.89	0.10	799.12
DY16	Gypenoside XXXV	90069-02-6	5.85	0.04	444.77
DY17	Gypenoside XXVII_qt	81474-82-0	30.21	0.74	418.73
DY18	Gypenoside XXVIII_qt	81474-80-8	32.08	0.74	416.71
DY19	Gypenoside XXXII	176182-04-0	34.24	0.25	787.11
DY20	Gypentonoside A_qt	157752-01-7	36.13	0.80	472.78
DY21	Pyrocatechuic acid	875-28-5	28.55	0.04	176.102
DY22	8-Hydroxyquinoline	148-24-3	NA	NA	145.158
DY23	7,8-Dihydroxycoumarin	486-35-1	NA	NA	178.14
DY24	Nicotiflorin	6130-58-1	3.64	0.73	594.518
DY25	Kaempferol 3-Glucorhamnoside	40437-72-7	NA	NA	594.518
DY26	Ginsenoside Rg1	22427-39-0	NA	NA	801.013
DY27	Gypenoside XVII	80321-69-3	3.51	0.10	947.154
DY28	Ginkgolic acid (C13:0)	20261-38-5	NA	NA	320.466
DY29	Ginkgolic Acid (C15:1)	22910-60-7	NA	NA	306.504
DY30	Isorhamnetin-3-O-nehesperidine	55033-90-4	NA	NA	624.54
DY31	Isoquercitrin	21637-25-2	1.86	0.77	464.094
DY32	Dulcitol	608-66-2	NA	NA	182.172

NA indicates that the data were not detected.

## Data Availability

Data are contained within the article.

## References

[1] Ogle G.D., James S., Dabelea D., Pihoker C., Svennson J., Maniam J., Klatman E.L., Patterson C.C. (2022). Global estimates of incidence of type 1 diabetes in children and adolescents: Results from the International Diabetes Federation Atlas, 10th edition. Diabetes Res. Clin. Pract..

[2] Zheng Y., Ley S.H., Hu F.B. (2018). Global aetiology and epidemiology of type 2 diabetes mellitus and its complications. Nat. Rev. Endocrinol..

[3] Li F., Yang F., Liu X., Wang L., Chen B., Li L., Wang M. (2017). Cucurbitane glycosides from the fruit of Siraitia grosvenori and their effects on glucose uptake in human HepG2 cells in vitro. Food Chem..

[4] Wang Z., Zhao X., Liu X., Lu W., Jia S., Hong T., Li R., Zhang H., Peng L., Zhan X. (2019). Anti-diabetic activity evaluation of a polysaccharide extracted from *Gynostemma pentaphyllum*. Int. J. Biol. Macromol..

[5] Chen J., Ning C., Mu J., Li D., Ma Y., Meng X. (2021). Role of Wnt signaling pathways in type 2 diabetes mellitus. Mol. Cell. Biochem..

[6] Vijan S. (2020). Type 2 Diabetes. Ann. Intern. Med..

[7] Laakso M. (2019). Biomarkers for type 2 diabetes. Mol. Metab..

[8] Yang C., Liu H., Li X., Peng X., Rao G., Xie Z., Yang Q., Du L., Xie C. (2023). Modular characteristics and mechanism of action of herbs for type 2 diabetes treatment in Chinese medicine. Heliyon.

[9] Di S., Han L., Zhao L., Tong X. (2019). Exploring the Mechanisms of Berberine against Type 2 Diabetes Mellitus and Its Complications by Network Pharmacology. Diabetes.

[10] Huang Y.-P., Wang Y.-S., Liu Y.-Y., Jiang C.-H., Wang J., Jiang X.-Y., Liu B.-W., Wang L., Ye W.-C., Zhang J. (2022). Chemical Characterization and Atherosclerosis Alleviation Effects of Gypenosides from *Gynostemma pentaphyllum* through Ameliorating Endothelial Dysfunction via the PCSK9/LOX-1 Pathway. J. Agric. Food Chem..

[11] Xie P., Luo H.-T., Pei W.-J., Xiao M.-Y., Li F.-F., Gu Y.-L., Piao X.-L. (2024). Saponins derived from *Gynostemma pentaphyllum* regulate triglyceride and cholesterol metabolism and the mechanisms: A review. J. Ethnopharmacol..

[12] Su C., Li N., Ren R., Wang Y., Su X., Lu F., Zong R., Yang L., Ma X. (2021). Progress in the Medicinal Value, Bioactive Compounds, and Pharmacological Activities of *Gynostemma pentaphyllum*. Molecules.

[13] Huyen V.T.T., Phan D.V., Thang P., Hoa N.K., Ostenson C.G. (2010). Antidiabetic Effect of *Gynostemma pentaphyllum* Tea in Randomly Assigned Type 2 Diabetic Patients. Horm. Metab. Res..

[14] Norberg A., Hoa N.K., Liepinsh E., Van Phan D., Thuan N.D., Jornvall H., Sillard R., Ostenson C.-G. (2004). A novel insulin-releasing substance, phanoside, from the plant *Gynostemma pentaphyllum*. J. Biol. Chem..

[15] Hoa N.K., Norberg A., Sillard R., Van Phan D., Thuan N.D., Dzung D.T.N., Jornvall H., Ostenson C.-G. (2007). The possible mechanisms by which phanoside stimulates insulin secretion from rat islets. J. Endocrinol..

[16] Megalli S., Davies N.M., Roufogalis B.D. (2006). Anti-hyperlipidemic and hypoglycemic effects of *Gynostemma pentaphyllum* in the Zucker fatty rat. J. Pharm. Pharm. Sci. A Publ. Can. Soc. Pharm. Sci. Soc. Can. Des Sci. Pharm..

[17] Ji X., Shen Y., Guo X. (2018). Isolation, Structures, and Bioactivities of the Polysaccharides from *Gynostemma pentaphyllum* (Thunb.) Makino: A Review. Biomed Res. Int..

[18] Yu Z., Zhang W., Li B., Bao P., Wang F., Sun J., Song G., Yin L., Nan Z. (2021). Efficacy and safety of acupuncture combined with Chinese Herbal Medicine for diabetic nephropathy A protocol for systematic review and meta-analysis. Medicine.

[19] Lokman E.F., Gu H.F., Mohamud W.N.W., Ostenson C.-G. (2015). Evaluation of Antidiabetic Effects of the Traditional Medicinal Plant *Gynostemma pentaphyllum* and the Possible Mechanisms of Insulin Release. Evid.-Based Complement. Altern. Med..

[20] Casas A.I., Hassan A.A., Larsen S.J., Gomez-Rangel V., Elbatreek M., Kleikers P.W.M., Guney E., Egea J., Lopez M.G., Baumbach J. (2019). From single drug targets to synergistic network pharmacology in ischemic stroke. Proc. Natl. Acad. Sci. USA.

[21] Doncheva N.T., Morris J.H., Gorodkin J., Jensen L.J. (2019). Cytoscape StringApp: Network Analysis and Visualization of Proteomics Data. J. Proteome Res..

[22] Agu P.C., Afiukwa C.A., Orji O.U., Ezeh E.M., Ofoke I.H., Ogbu C.O., Ugwuja E.I., Aja P.M. (2023). Molecular docking as a tool for the discovery of molecular targets of nutraceuticals in diseases management. Sci. Rep..

[23] Zheng G., Gan L., Jia L.-Y., Zhou D.-C., Bi S., Meng Z.-Q., Guan G.-J., Huang M.-M., He X., Zhang C.-F. (2021). Screen of anti-migraine active compounds from Duijinsan by spectrum-effect relationship analysis and molecular docking. J. Ethnopharmacol..

[24] Xia T., Duan W., Zhang Z., Fang B., Zhang B., Xu B., de la Cruz C.B.V., El-Seedi H., Simal-Gandara J., Wang S. (2021). Polyphenol-rich extract of Zhenjiang aromatic vinegar ameliorates high glucose-induced insulin resistance by regulating JNK-IRS-1 and PI3K/Akt signaling pathways. Food Chem..

[25] Qi B., Ren D., Li T., Niu P., Zhang X., Yang X., Xiao J. (2022). Fu Brick Tea Manages HFD/STZ-Induced Type 2 Diabetes by Regulating the Gut Microbiota and Activating the IRS1/PI3K/Akt Signaling Pathway. J. Agric. Food Chem..

[26] Zhang Y., Yang J.-H. (2013). Activation of the PI3K/Akt Pathway by Oxidative Stress Mediates High Glucose-Induced Increase of Adipogenic Differentiation in Primary Rat Osteoblasts. J. Cell. Biochem..

[27] Liu T.-Y., Shi C.-X., Gao R., Sun H.-J., Xiong X.-Q., Ding L., Chen Q., Li Y.-H., Wang J.-J., Kang Y.-M. (2015). Irisin inhibits hepatic gluconeogenesis and increases glycogen synthesis via the PI3K/Akt pathway in type 2 diabetic mice and hepatocytes. Clin. Sci..

[28] He L., Li Y., Zeng N., Stiles B.L. (2020). Regulation of basal expression of hepatic PEPCK and G6Pase by AKT2. Biochem. J..

[29] Wang M., Chen M., Guo R., Ding Y., Zhang H., He Y. (2022). The improvement of sulforaphane in type 2 diabetes mellitus (T2DM) and related complications: A review. Trends Food Sci. Technol..

[30] Acquah C., Dzuvor C.K.O., Tosh S., Agyei D. (2022). Anti-diabetic effects of bioactive peptides: Recent advances and clinical implications. Crit. Rev. Food Sci. Nutr..

[31] Lundqvist L.C.E., Rattigan D., Ehtesham E., Demmou C., Ostenson C.-G., Sandstrom C. (2019). Profiling and activity screening of Dammarane-type triterpen saponins from *Gynostemma pentaphyllum* with glucose-dependent insulin secretory activity. Sci. Rep..

[32] Merovci A., Abdul-Ghani M., Mari A., Solis-Herrera C., Xiong J., Daniele G., Tripathy D., DeFronzo R.A. (2016). Effect of Dapagliflozin With and Without Acipimox on Insulin Sensitivity and Insulin Secretion in T2DM Males. J. Clin. Endocrinol. Metab..

[33] Siddiqui S.A., Khan S., Wani S.A. (2022). Controlling diabetes with the aid of medicinal herbs: A critical compilation of a decade of research. Crit. Rev. Food Sci. Nutr..

[34] Liu Y., Qiu Y., Chen Q., Han X., Cai M., Hao L. (2021). Puerarin suppresses the hepatic gluconeogenesis via activation of PI3K/Akt signaling pathway in diabetic rats and HepG2 cells. Biomed. Pharmacother..

[35] He C.-J., Ma L.-Q., Iqbal M.S., Huang X.-J., Li J., Yang G.-Z., Ihsan A. (2020). Veratrilla baillonii Franch exerts anti-diabetic activity and improves liver injury through IRS/PI3K/AKT signaling pathways in type 2 diabetic db/db mice. J. Funct. Foods.

[36] Hosomi K., Saito M., Park J., Murakami H., Shibata N., Ando M., Nagatake T., Konishi K., Ohno H., Tanisawa K. (2022). Oral administration of Blautia wexlerae ameliorates obesity and type 2 diabetes via metabolic remodeling of the gut microbiota. Nat. Commun..

[37] Man X., Sai Z. (2022). Study on the Prognosis Effect of Traditional Chinese Medicine Treatment in DR Patients Based on the Perspective of Network Pharmacology. Contrast Media Mol. Imaging.

[38] Phi Hung N., Gauhar R., Hwang S.L., Trong Tuan D., Park D.C., Kim J.E., Song H., Huh T.L., Oh W.K. (2011). New dammarane-type glucosides as potential activators of AMP-activated protein kinase (AMPK) from *Gynostemma pentaphyllum*. Bioorganic Med. Chem..

[39] Song M., Tan D., Li B., Wang Y., Shi L. (2022). Gypenoside ameliorates insulin resistance and hyperglycemia via the AMPK-mediated signaling pathways in the liver of type 2 diabetes mellitus mice. Food Sci. Hum. Wellness.

[40] Chen H., Cai Y., Wu W.X. (2023). Analysis on the improvement of blood sugar and myocardial cells in patients with type 2 diabetes treated with *Gynostemma pentaphyllum* combined with repaglinide. Diabetes New World.

[41] Yeo J., Kang Y.-J., Jeon S.-M., Jung U.J., Lee M.-K., Song H., Choi M.-S. (2008). Potential hypoglycemic effect of an ethanol extract of *Gynostemma pentaphyllum* in C57BL/KsJ-db/db mice. J. Med. Food.

[42] Gao D., Zhao M., Qi X., Liu Y., Li N., Liu Z., Bian Y. (2016). Hypoglycemic effect of *Gynostemma pentaphyllum* saponins by enhancing the Nrf2 signaling pathway in STZ-inducing diabetic rats. Arch. Pharmacal Res..

[43] Elmoghayer M.E., Saleh N.M., Abu Hashim I.I. (2023). Enhanced oral delivery of hesperidin-loaded sulfobutylether-β-cyclodextrin/chitosan nanoparticles for augmenting its hypoglycemic activity: In vitro-in vivo assessment study. Drug Deliv. Transl. Res..

[44] Lee D., Kim J.-Y., Kwon H.C., Kwon J., Jang D.S., Kang K.S. (2022). Dual Beneficial Effects of α-Spinasterol Isolated from Aster pseudoglehnii on Glucose Uptake in Skeletal Muscle Cells and Glucose-Stimulated Insulin Secretion in Pancreatic β-Cells. Plants.

[45] Zhang L., Pan M.-Y., Li T., Jin Z.-M., Liu Z., Liu Q.-Y., Liu Y., Ding J.-Y., Jiang H., Hou X. (2023). Study on Optimal Extraction and Hypoglycemic Effect of Quercetin. Evid.-Based Complement. Altern. Med. Ecam.

[46] Wang J., Yu Y., Zhang H., Li L., Wang J., Su S., Zhang Y., Song L., Zhou K. (2024). Gypenoside XVII attenuates renal ischemia-reperfusion injury by inhibiting endoplasmic reticulum stress and NLRP3 inflammasome-triggered pyroptosis. Eur. J. Pharmacol..

[47] Zhou Y., Wu R., Cai F.-F., Zhou W.-J., Lu Y.-Y., Zhang H., Chen Q.-L., Su S.-B. (2021). Xiaoyaosan decoction alleviated rat liver fibrosis via the TGFβ/Smad and Akt/FoxO3 signaling pathways based on network pharmacology analysis. J. Ethnopharmacol..

[48] Xiao Y., Liu Y., Lai Z., Huang J., Li C., Zhang Y., Gong X., Deng J., Ye X., Li X. (2021). An integrated network pharmacology and transcriptomic method to explore the mechanism of the total Rhizoma Coptidis alkaloids in improving diabetic nephropathy. J. Ethnopharmacol..

[49] Wang H., Tan H., Zhan W., Song L., Zhang D., Chen X., Lin Z., Wang W., Yang Y., Wang L. (2021). Molecular mechanism of Fufang Zhenzhu Tiaozhi capsule in the treatment of type 2 diabetes mellitus with nonalcoholic fatty liver disease based on network pharmacology and validation in minipigs. J. Ethnopharmacol..

[50] An W., Huang Y., Chen S., Teng T., Shi Y., Sun Z., Xu Y. (2021). Mechanisms of Rhizoma Coptidis against type 2 diabetes mellitus explored by network pharmacology combined with molecular docking and experimental validation. Sci. Rep..

[51] Himanshu D., Ali W., Wamique M. (2020). Type 2 diabetes mellitus: Pathogenesis and genetic diagnosis. J. Diabetes Metab. Disord..

[52] Li D., Yang Y., Sun L., Fang Z., Chen L., Zhao P., Wang Z., Guo Y. (2020). Effect of young apple (Malus domestica Borkh. cv. Red Fuji) polyphenols on alleviating insulin resistance. Food Biosci..

[53] Gong P., Xiao X., Wang S., Shi F., Liu N., Chen X., Yang W., Wang L., Chen F. (2023). Hypoglycemic effect of astragaloside IV via modulating gut microbiota and regulating AMPK/SIRT1 and PI3K/AKT pathway (vol 281, 114558, 2021). J. Ethnopharmacol..

[54] Wang J., Wu T., Fang L., Liu C., Liu X., Li H., Shi J., Li M., Min W. (2020). Anti-diabetic effect by walnut (*Juglans mandshurica* Maxim.)-derived peptide LPLLR through inhibiting α-glucosidase and α-amylase, and alleviating insulin resistance of hepatic HepG2 cells. J. Funct. Foods.

[55] Nakayama M., Hisatsune J., Yamasaki E., Isomoto H., Kurazono H., Hatakeyama M., Azuma T., Yamaoka Y., Yahiro K., Moss J. (2009). Helicobacter pylori VacA-induced Inhibition of GSK3 through the PI3K/Akt Signaling Pathway. J. Biol. Chem..

[56] Nakae J., Biggs W.H., Kitamura T., Cavenee W.K., Wright C.V.E., Arden K.C., Accili D. (2002). Regulation of insulin action and pancreatic beta-cell function by mutated alleles of the gene encoding forkhead transcription factor Foxo1. Nat. Genet..

[57] Webb A.E., Brunet A. (2014). FOXO transcription factors: Key regulators of cellular quality control. Trends Biochem. Sci..

[58] Lv Q., Shi J., Miao D., Tan D., Zhao C., Xiong Z., Zhang X. (2024). miR-1182-mediated ALDH3A2 inhibition affects lipid metabolism and progression in ccRCC by activating the PI3K-AKT pathway. Transl. Oncol..

[59] Zhu J., Wu M., Zhou H., Cheng L., Wei X., Wang Y. (2021). Liubao brick tea activates the PI3K-Akt signaling pathway to lower blood glucose, metabolic disorders and insulin resistance via altering the intestinal flora. Food Res. Int..

[60] Huang X., Liu G., Guo J., Su Z. (2018). The PI3K/AKT pathway in obesity and type 2 diabetes. Int. J. Biol. Sci..

